# An ID-Associated Application to Facilitate Patient-Tailored Management of Multiple Sclerosis

**DOI:** 10.3390/brainsci11081061

**Published:** 2021-08-12

**Authors:** Michael Lang, Daniela Rau, Lukas Cepek, Fia Cürten, Stefan Ringbauer, Martin Mayr

**Affiliations:** 1 NeuroPoint Patientenakademie, 89073 Ulm, Germany; rau@neuropoint.de (D.R.); cepek@neuropoint.de (L.C.); 2 NeuroSys GmbH, 89081 Ulm, Germany; fia.cuerten@neurosys.de (F.C.); stefan.ringbauer@neurosys.de (S.R.); martin.mayr@neurosys.de (M.M.)

**Keywords:** multiple sclerosis, chronic disease, disease management, Patient Reported Outcomes, e-health, app, communication, digital tools

## Abstract

Despite improvements in diagnosis and treatment, multiple sclerosis (MS) is the leading neurological cause of disability in young adults. As a chronic disease, MS requires complex and challenging management. In this context, eHealth has gained an increasing relevance. Here, we aim to summarize beneficial features of a mobile app recently implemented in clinical MS routine as well as beyond MS. PatientConcept is a CE-certified, ID-associated multilingual software application allowing patients to record relevant health data without disclosing any identifying data. Patients can voluntarily share their health data with selected physicians. Since its implementation in 2018, about 3000 MS patients have used PatientConcept. Initially developed as a physician–patient communication platform, the app maps risk management plans of all current disease modifying therapies and thereby facilitates adherence to specified monitoring appointments. It also allows continuous monitoring of various PROs (Patient Reported Outcomes), enabling a broad overview of the disease course. In addition, various studies/projects currently assess monitoring, follow-up, diagnostics and telemetric evaluations of patients with other diseases beyond MS. Altogether, PatientConcept offers a broad range of possibilities to support physician–patient communication, implementation of risk management plans and assessment of PROs. It is a promising tool to facilitate patient-tailored management of MS and other chronic diseases.

## 1. Introduction

“Every 5 min, someone, somewhere in the world is diagnosed with MS” [1]. As the leading neurological cause of disability in young adults [1], MS is considered a growing global problem [2,3]. Disease symptoms are highly individual, ranging from fatigue, visual and bladder disorders, spasticity and mobility restriction to psychological disorders such as depression [4,5] which have a severe impact on the patient’s life.

Despite improvements in the diagnosis of MS and a major expansion of effective treatment options [2], disease management remains complex and challenging. As with other chronic diseases, it usually requires long periods of supervision, including regular monitoring and routine visits to observe disease progress or complications [6]. Therefore, there is a high demand for individually tailored concepts that facilitate patients’ access to healthcare and support the treating physicians in the provision of healthcare services.

During recent years, the development of digital healthcare technologies including mobile phone apps has gained an increasing relevance in disease management [7,8,9,10]. In particular for complex and unpredictable diseases such as MS, eHealth can improve monitoring and individual treatment [11], and furthermore promises to expand the possibilities for patients to manage their own care [12]. The relevance of eHealth is also underlined by the German Digital Healthcare Act, a recently passed law to support the future use of apps and other digital applications in the health system [13].

We successfully implemented an ID-associated application to facilitate patient-tailored management of MS [14]. Here, we aim to summarize its features in clinical routine with MS patients as well as its use in projects beyond MS, demonstrating the broad spectrum of possible applications and thus its adaptability to different diseases and individual patient needs.

## 2. Materials

The PatientConcept app is a CE (Conformité Européenne) certified mobile health software application that was developed in 2016 by a multidisciplinary team of physicians (neurologists, psychiatrists, experts on diabetes) and statisticians in cooperation with an IT company. Development was conducted in compliance with essential health and safety requirements of Directive 93/42/EEC and the product-related harmonized standards and specifications DIN EN ISO 14971, DIN EN ISO 62304, IEC 62366 and DIN EN ISO 13485 [14]. To ensure data safety, the app employs a secure ID associated data management, in which each patient receives their own, worldwide explicit ID for the patient’s mobile device. This approach allows distinguishing between medical data (e.g., blood values, disease history) and optional patient identifying data (optional details exclusively stored locally on the smartphone: name, phone number and date of birth). Thus, patients can record relevant health data without disclosing any identifying data. In case patients voluntarily decide to share their health data with selected physicians, practices and/or pharmacies by providing their personal ID, physicians can access health data and bidirectional communication is enabled.

The PatientConcept app is available for free download via the German app store (for both iOS and android smartphones or tablets). It can be used in a multilingual manner (German, English, Italian, French, Portuguese) [14].

## 3. Results

### 3.1. Application in the Field of MS

The app was initially developed as a doctor–patient communication platform with the aim of improving patient education, facilitating and thereby strengthening the communication between patients and their physicians, and to ease the daily routine of chronically ill patients. For example, the app provides a medication timer and simplifies requesting follow-up prescriptions for drugs and non-pharmacological therapies (physiotherapy, ergotherapy) from the treating physician with minimal effort.

The app enables bidirectional structured communication: Not only can patients contact their treating physician, but the physician can also communicate specifically with the patient. The patient can thereby receive regular information from the treatment center via push-news, e.g., on current seminars and disease-related updates and useful tips.

**Figure 1:**
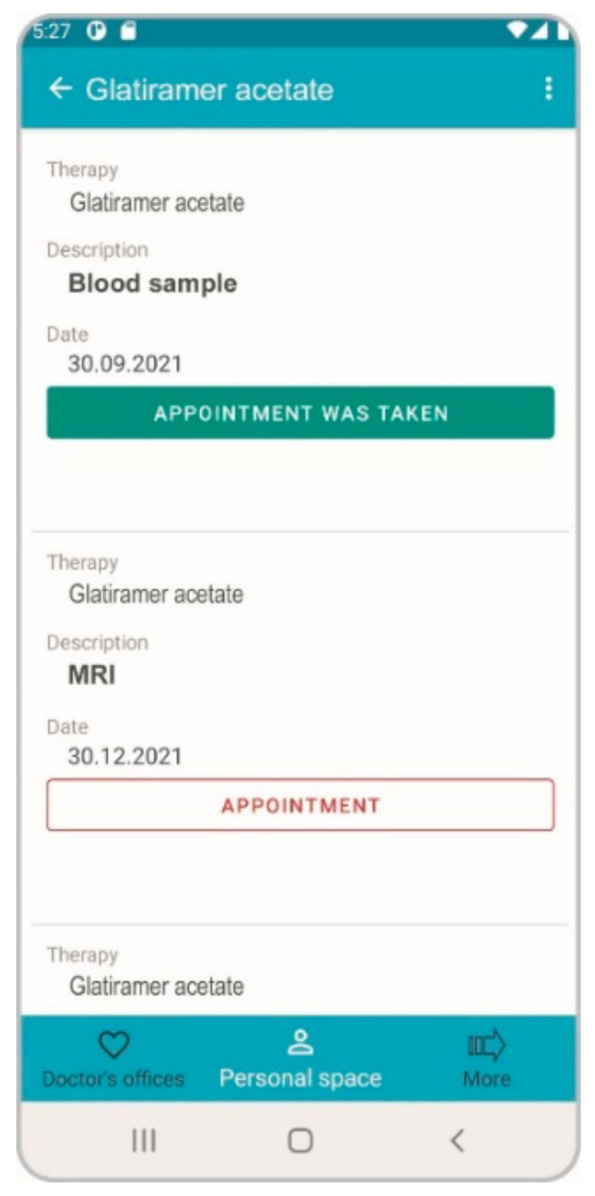
Risk Management Plan. PatientConcept currently maps all risk management plans in MS therapy. Control appointments are specified and checked by the system.

Since its implementation in 2018, about 3000 MS patients are/have been using the application. Meanwhile, many additional functions were added to the application. Risk management plans for all current disease modifying therapies have been integrated into the app to accompany the patient in adhering to the specified monitoring. In addition to reminding the patient of monitoring appointments (imaging, laboratory or consultative examinations) according to the guidelines of the respective therapy, the app also controls compliance. ToDo messages that have not been carried out or limit values that have been exceeded automatically result in specific information being sent to the responsible treatment practitioner (Figure 1).

**Figure 2:**
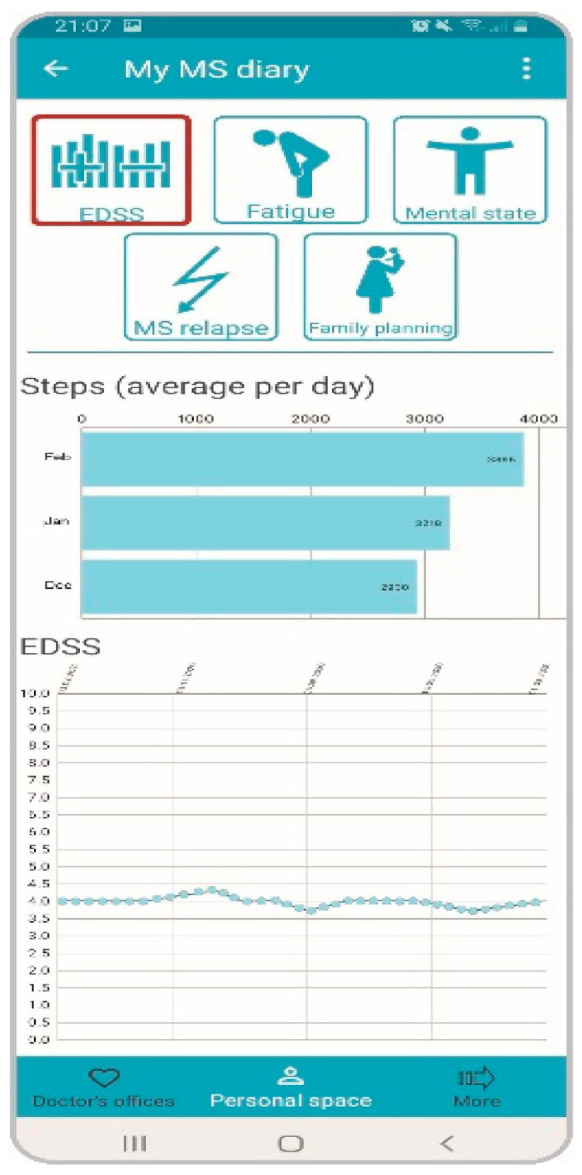
Monitoring of PROs. Patients complete tests/questionnaires on a regular basis, allowing monitoring of various patient reported outcomes.

**Figure 3:**
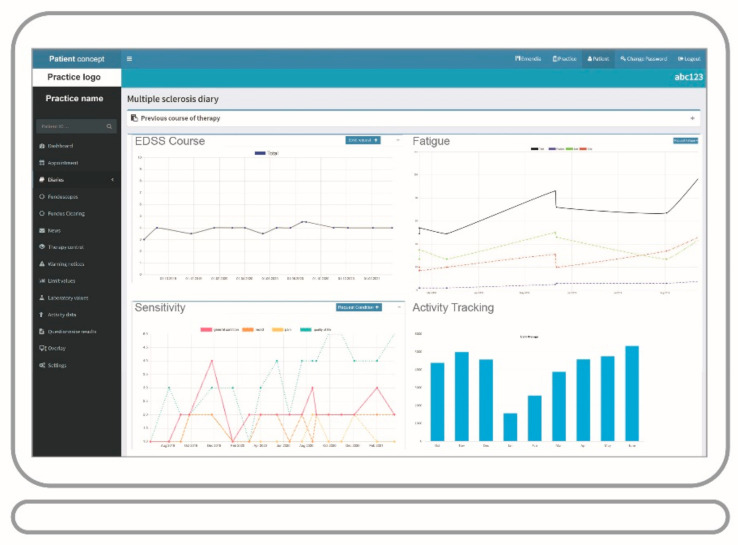
Treatment overview: Using a browser-supported portal for therapy monitoring, no additional software installation is necessary. The attending physician receives a comprehensive overview of the therapy course.

The app offers the possibility of entering various monitoring parameters, which are also checked by the system through an implemented red-flag warning system that can automatically inform the physician via red-flag email in case of critical data (e.g., aberrant blood values). In addition to the parameters specified in the risk management plan, the daily step count and important patient-reported outcome (PRO) measurements can be monitored (Figure 2) in PatientConcept and the upcoming MS-DiGA Emendia (described below). Examples include the assessment of cognition, pinching and drawing tests. Tests/questionnaires can be completed by the patients on a regular basis. Physicians receive supplementary information on the individual disease course via features such as the MS diary. Overall, physicians are provided with a broad treatment overview, e.g., on therapy history and disease progression (Figure 3).

Importantly, the app allows safe use across all indications. Through its anonymous ID-based data management system anonymous disease- and therapy-associated patient data can be collected over a prolonged period of time for research into therapy benefits and patient care.

Beyond the indication MS, the APP is used by about 1000 migraine patients and is currently also used in the indication of dizziness and gastroenterology, and furthermore for the follow-up of breast cancer patients in Germany and COVID patients in Luxembourg. In the meantime, the APP has been in sustained use in the indication of MS since its first publication 4 years ago, and there are more than 2 year data sets used in everyday clinical practice.

**Figure 4:**
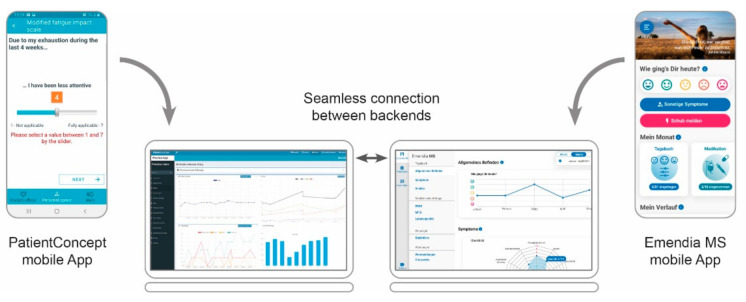
DiGA. Illustration of the interoperability of the PatientConcept and Emendia MS systems. A switch button enables seamless connection without additional log-in between the systems.

A new development for digital support of MS-therapy is the mobile health application Emendia (Latin: “emendare”—to improve), which is planned for market launch in Germany as a Digital Health Application (DiGA) in 2022. According to the German Digital Care Act, DiGAs can be prescribed by a physician at the expense of the statutory health insurance companies. To be approved as a DiGA, such an app has to comply with the strict medical guidelines of the Federal Institute for Drugs and Medical Devices. Emendia MS can provide opportunities for improved self-assessment of the patient’s disease status. This is accomplished by a combined collection of subjective parameters of the patient’s perceived well-being (e.g., self-perceived overall health) on the one hand and objective patient data (e.g., on motor function via sensors in the smartphone) on the other hand. In addition, Emendia MS aims to strengthen the health literacy of patients by providing valid, understandable information to help them better cope with their disease in everyday life. In contrast to the previously described system PatientConcept, which has been designed as a system for patient–physician communication, Emendia MS has been primarily created for individual use by the patient. Nevertheless, patients receive the opportunity to transmit their health data to the attending physician. In the case that the practice treats patients using either the PatientConcept or Emendia MS system, the backends of both systems are connected enabling interoperability. Patient searches across both systems via patient ID are made possible by this seamless connection without the need for an additional log-in (Figure 4).

### 3.2. Further Applications beyond MS

Besides MS, other chronic diseases also require permanent and continuous medical care. Therefore, we investigated whether the app is suitable for the management of patients with hereditary transthyretin-amyloidosis in an interdisciplinary setting (neurology/cardiology/gastroenterology, etc.) and according to GDPR (general data protection regulation). Time required for the consultation can be optimized, conversations are more specific and targeted, and the exchange between treating colleagues intensified [15].

Patients require monitoring not only during a disease, but also afterwards. The app is currently being used in a nationwide project in 40 cancer centres for continuous tumor follow-up of breast cancer patients (PRO B (Charite, Berlin); https://pro-b-projekt.de (accessed on 12 August 2021)). Corresponding projects are also planned for the follow-up care of patients with prostate carcinomas and testicular tumors.

In addition, the Luxemburg based CON-VINCE study (https://researchluxembourg.lu/covid-19-taskforce/con-vince, (accessed on 12 August 2021)) currently investigates the use of the app (available in five languages) in monitoring COVID patients across Luxembourg [16].

Beyond the aspect of monitoring, possibilities and limitations of the app as a diagnosis-supporting tool for the differentiation of selected neurological clinical pictures and disease groups were tested [17]. Diagnosis of rare neurological diseases is often difficult. Therefore, the aim was to use diagnosis-supporting procedures that recognise patterns in vast amounts of raw data via artificial intelligence (AI) and generate diagnostic suggestions based on these patterns. By means of specifically generated questionnaires, the app was able to support diagnostics through implemented differential diagnoses and could be used supportively for telemedical solutions (ARTIS Project— www.patientconcept.de/artis, (accessed on 12 August 2021)).

As possibilities in the telemetric evaluation of examinations are increasing, we recently tested the transmission of high-resolution images from the peripheral consultation to the ophthalmological specialist via PatientConcept. Analysis of 150 patients in five neurological practices demonstrated the basic feasibility of a telemetric assessment of ocular fundus images using the app [18].

For patients with Parkinson’s disease we also plan to launch a digital health application by the end of 2021/beginning of 2022. This results from further development of a project in which sensor data and diary entries from Parkinson’s patients were joint. The aim of the DiGA is to record the status of Parkinson’s disease and optimise therapeutic decisions.

## 4. Discussion

“The disease with a thousand faces” [19]—this well-known term for the chronic disease multiple sclerosis already implies the high and complex demands on disease management. One of the central aspects of disease management should be continuous and regular monitoring of safety [20]. Risk management plans define specific examinations for each drug; compliance with these is tedious as well as time- and cost-consuming and thus a major challenge for both patients and physicians. Nevertheless, implementation of risk management plans is indispensable to reduce or avoid serious side effects. To facilitate continuous monitoring, digital applications such as mobile apps have proven useful. In recent years, their development has advanced rapidly and can be of profound benefit [8,9]. mHealth (mobile Health) potentially may deliver healthcare regardless of time and place or geographical constraints [21,22]. Particularly for MS with its long and unpredictable disease course, long-term monitoring preferably with digital applications is a necessity [11,23]. Examples of apps supporting monitoring include the Novartis SymTrac app, My MS manager and MS dialog app [8]. The PatientConcept app and the integrated risk management plan can better help to comply with predefined and monitored requirements by reminding patients of their regular imaging, laboratory or consultation examinations and controlling for documented aberrant values.

Monitoring should not only contain safety aspects, but also clinical and subclinical disease activity to evaluate treatment effectiveness, supporting individualized treatment [20]. In addition to clinical and radiological monitoring, the patients themselves can contribute to evaluating the efficacy of their medication by documenting PRO measurements. These are provided directly by the patient and include symptoms, activity limitations, cognitive and health status, level of fatigue or quality of life [20,24]. Besides their relevance in clinical trials that increasingly define PROs as secondary or tertiary outcomes, PROs are playing an important role also in clinical practice in order to better understand the impact of MS and its therapy on the patient’s life under real world conditions [24,25].

Continuous assessment of PROs via the PatientConcept app enables the physician to follow the progression of the disease based on various parameters over time compared to the limited possibilities of a “snapshot” during the personal visit to the practice. It also saves valuable time that physicians could use more sensibly for their patients. In addition, continuous PRO monitoring facilitates the physician in discussing past processes and events with the patient more clearly. The immediate check of patient entries by the system, which informs of any aberrant values, allows the physician to intervene earlier and to make necessary treatment adjustments, resulting in a more patient-tailored therapy approach. The integrated walking assessment via step counts (by the smartphone) could be beneficial for evaluating the severity of the disease, as it facilitates a meaningful assessment of patient mobility [26]. PROs are one example of giving patients a voice. PREMs (patient-reported experience measures) should also be monitored long-term and integrated as a standard in order to identify and prevent problems at an early stage [26].

Patients should also be well informed and involved in treatment decisions to achieve patient engagement. Recognizing that each patient with their different needs is individual displays a core aspect of patient engagement [27], that results in better outcomes and treatment adherence [28,29,30,31]. Poor adherence displays a major challenge in MS management [32,33]. Adherence to prescribed disease-modifying treatments (DMTs) can be further improved by a better general connectivity of the patient to the practice or patient portal. Intensified support of MS patients via patient support programs (through patient portal, MS nurse or physicians) has been shown to be beneficial for improving quality of care, patients’ quality of life, patient participation and adherence in earlier studies [34,35,36,37,38,39]. Therefore, the app was initially developed to intensify bidirectional patient–physician communication and to increase patients’ commitment to the practice, thereby providing continuous support for chronically ill patients without burdening the physician’s time budget.

Since the Digital Health Care Act was introduced in December 2019, DiGAs are receiving growing attention. While a survey revealed that physicians perceive digital health applications as an opportunity, measures to increase acceptance particular among general practitioners appear sensible [40]. The presented DiGA Emendia is unique in allowing the patient to share health data with the treating physician, offering the advantage that a long-term use of Emendia for medical consultation and optimized therapy decisions could be ensured. Currently, 17 DIGAs are listed with others under review [41], aiming at further supporting patients in coping with their disease.

To reduce the patient’s burden, disease management should not only be tailored to the individual patient but should also include multidisciplinary assessment [42]. This aspect is of high relevance because a multimodal, interdisciplinary approach is indispensable for an effective management of multisymptomatic diseases such as MS and involves close and continuous collaboration between various specialists [43]. Applying such an interdisciplinary approach for MS patients has been shown to be difficult [44]. Since the parallel use of different apps is not expedient, a consolidated approach is desirable. While most devices are developed for use in the context of one specific disease, the PatientConcept platform (various complementary APPs but only one portal for the physician) can be employed across indications and thus allows easier interdisciplinary exchange and potentially improves the flow of information on medical content for the benefit of patients. This enables specific requests to a specialised centre even in structurally weak regions. Thus, the app might offer an opportunity to improve the quality of care and treatment and to increase the effectiveness of therapeutic measures in MS care. Particularly in times of a pandemic, continuous monitoring could be carried out with the help of the app, potentially supplying profound benefit.

Even though various mHealth (mobile Health) tools have been developed to ease disease management, monitoring, rehabilitation and education of MS patients [8], the usage of available medical software applications remains comparably low. Reasons include concerns about privacy and security [45]. To our knowledge, PatientConcept App was one of the first CE-certified apps and is utilizing a worldwide explicit ID used by the patient to allow safe and structured data management and transfer while ensuring the highest security standards and data privacy. This ID-based data management system could also provide large real-world data sets of anonymous disease-related and therapy-associated patient data over a prolonged period of time to evaluate therapy benefits and patient care outside of clinical trials.

## 5. Conclusions

The ID-associated CE-certified PatientConcept app is widely used, not only by MS patients, and offers a broad range of possibilities for enhancing bidirectional physician–patient communication, aiming at improving treatment adherence. The use of automated routines can facilitate the indispensable implementation of risk management plans. With its various adaptable features including the assessment of PROs and its possible implementation also in interdisciplinary approaches, the application is a promising tool to facilitate patient-tailored management of multiple sclerosis and other chronic diseases. The usage of PatientConcept with its secure data storage in daily patient care could also provide relevant data on the efficacy of medical measures in the real world.
